# A quantitative PCR method for measuring absolute telomere length

**DOI:** 10.1186/1480-9222-13-3

**Published:** 2011-01-31

**Authors:** Nathan J O'Callaghan, Michael Fenech

**Affiliations:** 1 CSIRO Food and Nutritional Sciences, Nutritional Genomics Laboratory, PO Box 10041, Adelaide BC, SA, 5000, Australia

## Abstract

We describe a simple and reproducible method to measure absolute telomere length (aTL) using quantitative real-time polymerase chain reaction (qPCR). This method is based on the Cawthon method for relative measurement of telomere length (TL) but modified by introducing an oligomer standard to measure aTL. The method describes the oligomer standards, the generation of the standard curve and the calculations required to calculate aTL from the qPCR data. The necessary controls and performance characteristics of the assay are described in detail and compared relative to other methods for measuring TL. Typical results for this assay for a variety of human tissue samples are provided as well as a troubleshooting schedule. This method allows high throughput measurement of aTL using small amounts of DNA making it amenable for molecular epidemiological studies. Compared to the traditional relative TL qPCR assays, the aTL method described in this protocol enables a more direct comparison of results between experiments within and between laboratories.

## Introduction

Telomeres are nucleoprotein structures that cap the ends of chromosomes. The integrity of the telomere structure and its DNA hexamer (TTAGGG)n repeat sequence is critical for the protection of the ends of chromosomes from degradation and in maintaining overall genomic stability[1,2]. The number of DNA hexamer (TTAGGG)n repeats is reduced during each cell division in differentiated cells, and as a consequence telomere length (TL) often decreases in most differentiated cells throughout the lifespan of the organism [1]. Shortening of telomeres can result in telomere end fusions and an increased level of chromosome instability (CIN), which is in turn a key initiating event in numerous cancers (including lung, breast, colon, and prostate cancers, as well as certain leukaemia's)[3-7]. It has been shown that telomere shortening can be accelerated by environmental factors such as psychological and physiological stress, cigarette smoking, obesity and high homocysteine [8-14]. Efficiency of TL maintenance is also affected by gender[15-17]. TL has been shown to be associated prospectively with increased risk of myocardial infarction, coronary artery disease, breast cancer free survival, clear cell renal cell carcinoma survival, post-stroke mortality, dementia and cognitive decline, as well as total survival independent of genetic influences [18-24].

For all of these reasons there has been a burgeoning interest in measuring TL accurately and efficiently to understand both the fundamental biology of telomere maintenance as well as determining the modifiable dietary and life-style factors that contribute substantially to accelerated TL attrition. A wide range of methods have been developed to measure TL such as (i) the gold standard Terminal Restriction Fragment (TRF) analysis by hybridisation of digested DNA with telomere sequence probes, (ii) Flow-FISH cytometry of cells following hybridisation with fluorescent peptide nucleic acid (PNA) probes, (iii) quantitative fluorescence *in situ *hybridisation (FISH) with fluorescent telomere PNA probes and (iv) qPCR assay. With the exception of the TRF assay all other methods have the disadvantage of generating a relative measure of TL. The advantage of the qPCR method is that, unlike the TRF assay only small amounts of DNA are required and can easily be performed in high-throughput format which is essential for large epidemiological studies [25,26].

The protocol we describe is a modification to Cawthon's qPCR based relative quantification (Telomere/Single Copy Gene ratio) method by introducing an oligomer standard to generate aTL values. The capability to generate aTL values allows a more direct comparison of results between experiments within and between laboratories. The protocol described here is for human buccal cells or isolated lymphocytes but can be easily adapted to other cell types or species.

## Experimental design

The measurement of aTL can be performed in any cell population from which high quality undamaged DNA can be collected. However the possibility of detecting meaningful differences between groups is likely to depend on the purity of the cell populations examined because of the possibility of different replicative histories which may affect TL. This is particularly true when doing *in vivo *studies with white blood cells because the ratios of lymphocytes, monocytes, basophils and granulocytes can vary greatly between individuals depending on their age and health condition(s). Therefore aTL should be measured in populations of single cell types as much as is practically possible. Other sources of variation could be the proportion of dead/dying cells which may have to be taken into consideration because necrotic or apoptotic cells may have a different TL from viable cells.

As indicated above there are an increasing demographic, nutritional and life-style variables and disease conditions associated with altered TL and these need to be considered when planning comparative *in vivo *studies. Below is a list of the key variables that should be considered in human studies of aTL [8-13,18-24,27-29]:

Date of Birth (include maternal and paternal age at birth)

Gender

History of cancer, cardiovascular disease and neurodegenerative disease

Inherited mutations that predispose to degenerative diseases listed above as well as accelerated ageing syndromes (e.g., WRN, ATM and BRCA1 mutations)

Medication or recent illness (disease history)

Smoking status and history

Exposure to chemical carcinogens and radiation e.g., X-ray

Indicators of psychological stress

Body Mass Index

Lifestyle index (e.g. physical activity, alcohol consumption, sunshine exposure)

Dietary habits measured using a validated food frequency questionnaire

Plasma concentration of B vitamins and homocysteine

Markers of oxidative stress (CRP, MDA etc)

Genotype

### Precautions regarding a qPCR based assay

Real-time quantitative PCR (qPCR) uses fluorescent signal detection to be monitored as the PCR reaction proceeds. This allows initial template levels to be precisely and accurately quantified. PCR is an extremely sensitive method of analysis and cross-contamination can lead to erroneous or false results. Therefore precautions and strict quality control ascertainment must be planned into to each and every experiment (further details can be found in [30]).

## Materials

### Reagents and Reagent Set-Up

#### Sample collection

##### Buccal cell buffer

Prepare 1 litre of the buccal buffer, [0.01 M Tris-hydrochloride (Sigma T-3253), 0.1 M ethylenediaminetetraacetic acid tetra Na salt (Sigma E5391), 0.02 M sodium chloride (Sigma S5886)]. Thoroughly dissolve the salts IN MilliQ water and make up the volume to 1000 ml. Adjust pH to 7.0 and autoclave at 121°C for 30 min. Buffer will last for up to 3 months when stored as a sterile solution in sealed bottles at room temperature.

Dimethyl sulphoxide (DMSO) (Sigma, hybrimax, sterile-filtered, #D2650).

Phosphate Buffered Saline (PBS)

Ficoll-Paque (GE Biosciences)

Hank's balanced salt solutions (HBSS)

Fetal bovine serum (FBS), heat-inactivated

#### DNA isolation

All solutions used for DNA isolation are purged with nitrogen (immediately prior to use) and supplemented with 50 μM phenyl-tert-butyl nitrone to minimise oxidative damage to DNA[31].

Qiagen DNAeasy Kit (#69506)

includes: Proteinase K, AW1, AW2, AE and Spin-columns

Dithiothreitol (DTT; Sigma)

Ethanol (Ajax Finechem #214-2.5L)

Nitrogen (for purging of buffers)

#### Oligomers (standards and primers)

CRITICAL: All oligomers should be HPLC purified; long oligomers (>50 mers) have a high failure rate during synthesis; this means there will be multiple failed sequences which must be removed in order to maintain accuracy of oligomer standards (GeneWorks, Adelaide). All oligomers are diluted in appropriate volume of PCR grade water and stored at -20degC until required. Working stocks of oligomers should be made fresh; dilutions are stable at 4degC for up to 2 weeks. Oligomer sequences are shown in Table 1.

**Table 1:** Oligomers used for aTL assay in human and rodent.

	Oligomer Name	Species	Oligomer sequence (5'-3')	Amplicon size
**Standards**	Telomere standard	Human/rodent	(TTAGGG)14	**84 bp**
	
	36B4 standard	Human	CAGCAAGTGGGAAGGTGTAATCCGTCTCCACAGACAAGGCCAGGACTCGTTTGTACCCGTTGATGATAGAATGGG	**75 bp**

**PCR Primers**	teloF	Human/rodent	CGGTTTGTTTGGGTTTGGGTTTGGGTTTGGG TTTGGGTT	**>76 bp**
		
	teloR	Human/rodent	GGCTTGCCTTACCCTTACCCTTACCC TTACCCTTACCCT	
	
	*36B4F*	Human	CAGCAAGTGGGAAGGTGTAATCC	**75 bp**
		
	*36B4R*	Human	CCCATTCTATCATCAACGGGTACAA	
	
	*b-globinF*	Human	GCTTCTGACACAACTGTGTTCACTAGC	**82 bp**
		
	*b-globinR*	Human	CACCAACTTCATCCACGTTCACC	
	
	*36B4F*	Rodent	ACTGGTCTAGGACCCGAGAAG	**78 bp**
		
	***36B4R***	**Rodent**	**TCAATGGTGCCTCTGGAGATT**	

### Standard Curves and associated calculations

#### Telomere Standard Curve

A standard curve is established by dilution of known quantities of a synthesised 84 mer oligonucleotide containing only TTAGGG repeats. The number of repeats in each standard is calculated using standard techniques as follows:

• The oligomer standard is 84 bp in length (TTAGGG repeated 14 times), with a molecular weight (MW) of 26667.2.

• The weight of one molecule is MW/Avogadro's number. Therefore, weight of telomere standard is: 2.6667 × 10^4^/6.02 × 10^23 ^= 0.44 × 10^-19^g.

• The highest concentration standard (TEL STD A) has 60 pg of telomere oligomer (60 × 10^-12^g) per reaction.

• Therefore there are 60 × 10^-12^/0.44 × 10^-19 ^= 1.36 × 10^9 ^molecules of oligomer in TEL STD A.

• The amount of telomere sequence in TEL STD A is calculated as: 1.36 × 10^9 ^× 84 (oligomer length) = 1.18 × 10^8 ^kb of telomere sequence in TEL STD A.

A standard curve is generated by performing the aTL qPCR assay on serial dilutions of TEL STD A (10^-1 [^1.18 × 10^8^] through to 10^-6 ^[1.18 × 10^3^] dilution).

**Figure 1:**
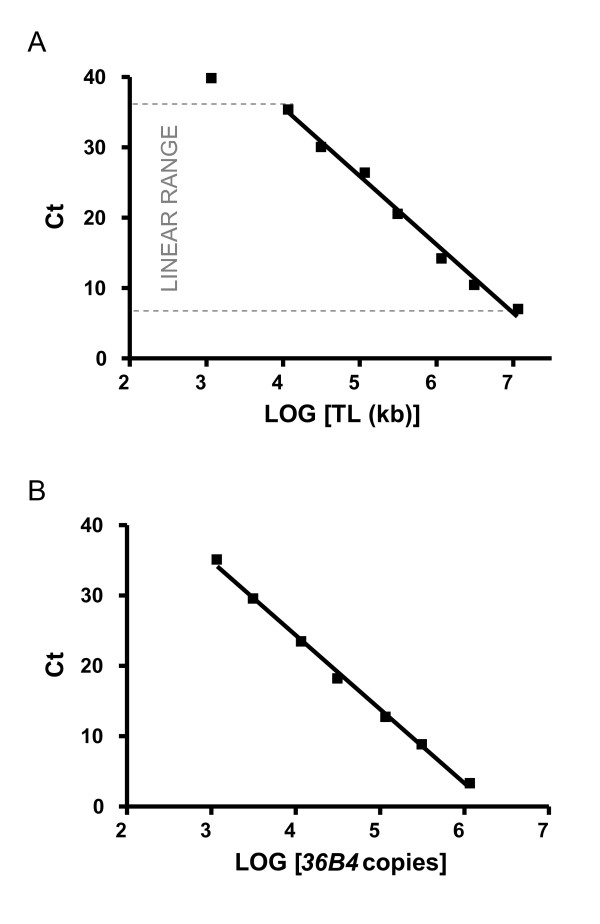
**Standard curve used to calculate absolute telomere length**. C_T _(cycle threshold) is the number of PCR cycles for which enough SYBR green fluorescence was detected above background. A) Graph shows standard curve for calculating length of telomere sequence per reaction tube. X-axis represents amount of telomere sequence in kb per reaction. Correlation coefficient within the linear range was 0.98. The graph shown here represents the linear range of the PCR. The DNA amount was optimised so that experimental samples are detected within the linear range. The value generated from the experimental samples utilising this standard curve was equal to kb of telomere sequence per sample. B) Graph shows standard curve for calculating genome copies using *36B4 *copy number. Correlation coefficient was 0.99. Standard curves were generated using an AB 7300 Sequence Detection System with the SDS Ver. 1.9 software (Applied Biosystems, Foster City, CA).

Plasmid DNA (*pBR322*) is added to each standard to maintain a constant 20 ng of total DNA per reaction tube. The standard curve was used to measure content of telomeric sequence per sample in kb (Figure 1).

#### Single copy gene (SCG) standard

A single copy gene (SCG) is used as a control for amplification for every sample performed and to determine genome copies per sample. The choice of SCG is critical for reliability of results; any change in copy number can substantially impact upon aTL measurements. We routinely use *36B4*, which encodes the acidic ribosomal phosphoprotein P0; *b-globin *is also frequently used. NOTE: Remember that although telomeric DNA sequence is consistent in mammals, the SCG will be different, thus a SCG standard curve and amplicon must be generated for each target species.

CRITICAL: SCG amplification is crucial for the accuracy of the results generated in the aTL qPCR assay; changes in amount of template that is present in each reaction can by affected by pipetting or DNA quantification error. Variation in SCG copy number (CNV) may also occur between individuals and between normal and cancerous cells; CNV can be assessed by using multiple SCG amplicons to generate diploid copies per PCR reaction.

#### Standard curve for human SCG, 36B4

Genome copy number per reaction is calculated as follows:

• The synthesised *36B4 *oligomer standard is 75 bp in length with a MW of 23268.1.

• The weight of one molecule is MW/Avogadro's number. Therefore, weight of the synthesised *36B4 *oligomer standard is: 2.32681 × 10^4^/6.02 × 10^23 ^= 0.38 × 10^-19^g.

• The highest concentration standard (SCG STD A) had 200 pg of *36B4 *oligomer (200 × 10^-12^g) per reaction.

• Therefore there are 200 × 10^-12^/0.38 × 10^-19 ^= 5.26 × 10^9 ^copies of *36B4 *amplicon in SCG STD A.

• Therefore SCG STD A is equivalent to 2.63 × 10^9 ^diploid genome copies, because there are two copies of *36B4 *per diploid genome.

A standard curve was generated by performing serial dilutions of SCG STD A (10^-1 ^through to 10^-6^dilution).

Plasmid DNA (*pBR322*) was added to each standard to maintain a constant 20 ng of total DNA per reaction tube. The standard curve was used to measure diploid genome copies per sample (Figure 1).

### PCR

Power SYBR I mastermix (Applied Biosystems, #4367396). Contains AmpliTaq Gold DNA polymerase, dNTPs, SYBR I Green Dye, opitimised buffers and passive reference dye (ROX) - CAUTION SYBR I Green dye may be carcinogenic when ingested or absorbed into skin. Wear appropriate gloves when working with this solution.

PCR Grade water

Long telomere positive control DNA (cell line 1301; accession number 01051619, European Collection of Cell Cultures, UK EQUIPMENT)

Plasmid DNA *PBR322 *(Sigma)

### Equipment

#### Sample collection

Biological safety cabinet.

Bench top centrifuge, biosafe.

Haemocytometer

30 ml yellow topped polystyrene containers (Sarstedt #60.9922.918).

10 ml graduated sterile pipettes (Falcon #7551).

Milli-Q water (Milli-Q water purification system, Adelab Scientific, SA).

Sterile plugged Pasteur pipettes 900 (22-23 cm) (Chase #93P).

Syringes 10 ml (Crown Scientific #SS + 10S).

Needles 18G (Crown Scientific # 2525RA).

##### Lymphocytes

Sterile vacutainer blood tubes with lithium heparin or EDTA (e.g. Greiner)

##### Buccal

Small headed toothbrushes (2 cm head length) (Supply SA, #85300012).

Nylon net filters 100 μm (Millipore, #MILNYH02500).

### DNA isolation

Benchtop micro-centrifuge

Nitrogen

Heat-block

1.5 ml microfuge tubes, DNAse free.

PCR plates and caps

Spectrophotometer for 260/280-nm measurements (i.e. NanoDrop, Biolabs)

### PCR

Pipettes (and tips)

Optical PCR plates and caps

Centrifuge for spinning plates

Real-Time PCR machine (e.g. Applied Biosystems 7300)

Software for analyzing data (e.g. Applied Biosystems SDS software v1.4)

Software for analyzing data (e.g. Microsoft Excel, SPSS)

## Procedure

### Sample collection

The measurement of aTL can be performed in any cell population from which high quality undamaged DNA can be collected. Below are some examples of collection procedures we routinely use in our research. All manipulations must be carried out in a Biological Safety Cabinet Class II.

#### Lymphocytes

I. Collect fresh blood by venipuncture into vacutainer blood tubes (LiHep or EDTA).

II. Dilute whole blood 1:1 with HBSS and gently invert to mix

III. Overlay diluted blood onto 1/3 volume of Ficoll-Paque being careful not to disturb the interface

IV. Centrifuge tubes at 400 g for 30 min at 20degC

V. Remove lymphocyte layer located at the interface of Ficoll-Paque and dilute plasma into a fresh tube using a Pasteur pipette.

VI. Dilute the cell suspension with 3x volume HBSS at room temperature

VII. Centrifuge at 180 g for 10 min

VIII. Discard supernatant and resuspend the cell pellet in HBSS.

IX. Perform cell count and viability test using haemocytometer and trypan blue

X. Centrifuge at 100 g for 10 min.

PAUSE POINT: Cells can be resuspended in 200 μl PBS and taken through to DNA isolation step below or if storage required proceed to step XI.

XI. Discard supernatant and resuspend the cell pellet in freezing media (FBS and 10%DMSO) resuspend at 5 × 10^6 ^cells/ml

XII. Place in Styrofoam box at -80degC.

XIII. Transfer to Liquid Nitrogen for long term storage until required

a. Cells can be stored for up to 10 years.

XIV. To thaw cells, remove from liquid nitrogen and place on ice

XV. Place vial in a beaker of 37degC water and agitate gently.

XVI. Once completely thawed, centrifuge vial for 5 min at 300 g.

XVII. Discard supernatant and resuspend pellet in 200 μl PBS

#### Buccal cells

I. Prior to buccal cell collection rinse mouth twice thoroughly with 100 ml of water.

II. Gently rotate a small-headed toothbrush (2 cm head length) 10 times firmly against the inside of the cheek wall in a circular motion.

III. The head of each brush is then placed into 10 ml of buffer and rotated repeatedly such that the cells are dislodged and released into buffer producing a cloudy suspension of buccal cells in the buffer.

IV. Centrifuge for ten minutes at 100 g.

V. Remove supernatant leaving approximately 1 ml of cell suspension and replace with another 5 ml of buccal buffer. Vortex briefly.

VI. Centrifuge at 100 g for ten minutes.

VII. Remove supernatant and resuspend in 5 mls of buccal buffer.

VIII. Vortex briefly and then homogenise for 2-3 minutes in a hand held tissue homogenizer to disaggregate cell clumps.

IX. Draw cells up into a syringe (with 18G needle) and pass through a 100 μm nylon filter into a fresh tube.

X. Centrifuge at 100 g for 10 mins and remove the supernatant.

XI. Resuspend in 1 ml of buccal cell buffer.

XII. Perform viable cell count using haemocytometer and trypan blue

XIII. Centrifuge at 100 g for 10 min.

PAUSE POINT: Cells can be resuspended in 200 μl PBS and taken through to DNA isolation or if storage required proceed to step XIV.

XIV. Discard supernatant and resuspend the cell pellet in freezing media (FBS and 10%DMSO) resuspend at 5 × 10^6 ^cells/ml

XV. Place in Styrofoam box at -80degC.

XVI. Transfer to Liquid Nitrogen for long term storage until required

a. Cells can be stored for up to 10 years.

XVII. To thaw cells, remove from liquid nitrogen and place on ice

XVIII. Place vial in a beaker of 37degC water and agitate gently.

XIX. Once completely thawed, centrifuge vial for 5 min at 300 g.

XX. Discard supernatant and resuspend pellet in 200 μl PBS

#### Tissue culture

I. Centrifuge appropriate number of cells for 5 min at 300 g.

PAUSE POINT: Cells can be resuspended in 200 μl PBS and taken through to DNA isolation or if storage required proceed to step XIV.

II. Discard supernatant and resuspend the cell pellet in freezing media (FBS and 10%DMSO) resuspend at 5 × 10^6 ^cells/ml

III. Place in Styrofoam box at -80degC.

IV. Transfer to Liquid Nitrogen for long term storage until required

a. Cells can be stored for up to 10 years.

V. To thaw cells, remove from liquid nitrogen and place on ice

VI. Place vial in a beaker of 37degC water and agitate gently.

VII. Once completely thawed, centrifuge vial for 5 min at 300 g.

VIII. Discard supernatant and resuspend pellet in 200 μl PBS

#### Other tissues (e.g. biopsy material)

I. Collect biopsy sample in 3 ml RPMI in a sterile tube.

II. Transfer sample to a new sterile tube containing 3 ml RPMI with forceps. Mix by inversion. Repeat twice.

III. Transfer sample to cryovial containing 900 μl FBS with forceps.

PAUSE POINT: Cells can be resuspended in 200 μl PBS and taken through to DNA isolation or if storage required proceed to step XIV.

IV. Discard supernatant and resuspend the cell pellet in freezing media (FBS and 10%DMSO) resuspend at 5 × 10^6 ^cells/ml

V. Place in Styrofoam box at -80degC.

VI. Transfer to Liquid Nitrogen for long term storage until required

a. Cells can be stored for up to 10 years.

VII. To thaw cells, remove from liquid nitrogen and place on ice

VIII. Place vial in a beaker of 37degC water and agitate gently.

IX. Once completely thawed, centrifuge vial for 5 min at 300 g.

X. Discard supernatant and resuspend pellet in 200 ul PBS

### DNA isolation

Prepare samples according to protocols above; you should have 200 μl of cell suspension in a microfuge tube. DNA isolation is performed as per manufacturer's directions (Qiagen, DNeasy Blood and Tissue Kit) with slight modifications.

1. Add 20 μl proteinase K (600 mAU/ml).

2. Add 180 μl Buffer AL (tissue and cell lysis buffer).

3. Mix by inversion 10-15 times.

4. Incubate at 37degC.

a. 3 hrs for lymphocytes and cultured cells

b. 6 hrs for tissue samples or until tissue is completely lysed

c. Optional - if tissue is not completely lysed add additional 200 μl Buffer AL

5. Add 200 μl Ethanol.

6. Mix by inversion.

7. Incubate at room temperature for 3 minutes.

8. Pipette mixture into spin column.

9. Centrifuge at >6000 × g for 1 minute.

10. Discard flow through.

11. Pipette 500 μl Buffer AW1 (wash buffer) into column.

12. Centrifuge at >6000 × g for 1 minute.

13. Discard flow through.

14. Pipette 500 μl Buffer AW2 (wash buffer) into column.

15. Centrifuge at >6000 × g for 1 minute.

16. Discard flow through.

17. Centrifuge at >6000 × g for 2 minutes.

18. Transfer spin column to clean microfuge tube.

19. Pipette 200 μl Buffer AE (elution buffer) into spin column.

20. Incubate at room temperature for 1 minute.

21. Centrifuge at >6000 × g for 2 minutes.

22. Pipette eluate back onto spin column.

23. Incubate at room temperature for 1 minute.

24. Centrifuge at >6000 × g for 2 minutes.

25. Add 1 mM DTT to eluate.

26. Store until required

PAUSE POINT.

a. store at 4degC if DNA to be used within 1 week

b. store at -20deg if DNA to be used within 2-6 weeks

c. store at -80deg if DNA is not to be used for 2 months

d. CRITICAL - minimise times stock DNA is freeze-thawed

27. DNA was quantified in triplicate using a NanoDrop spectrophotometer.

a. Yield and quality of DNA will vary depending on input material

b. 5 × 10^6 ^lymphocytes or cultured cells should yield 10-20 μg of DNA; 5 × 10^5 ^buccal cells approximately 1-5 μg DNA.

c. Pure DNA has a A260/280 ratio of 1.7-2.0

#### Normalisation plate

To reduce pipetting error in qPCR assay we generate a working concentration DNA plate. This plate includes DNA samples to be examined all at a consistent 5 ng/μl. Plan carefully the layout of this plate, to minimise effect of plate to plate variation, ensure samples from same individual are on a single plate

28. set up normalisation plate at 5 ng/μl

a. dilute concentrate DNA samples with PCR grade water (DNase/RNase Free) into 96-well plate

b. make enough to complete required PCR reactions (minimum 50 μl - maximum 200 μl)

29. Store plate at 4degC until required

a. Diluted DNA can be stored up to 1 year at 4degC

### qPCR for aTL

All samples are run on an ABI 7300 Sequence Detection System with the SDS Ver. 1.9 software (Applied Biosystems [AB] Foster City, CA).

30. Prepare master mix solution (Table 2). Prepare enough for samples to be run in triplicate, no template control (NTC), positive control (1301 cell line DNA), standards (which should be run on every plate) plus an extra 5% for pipetting error.

**Table 2:** Representation of master mix preparation.

Reagents	Volumes for one sample (ul)	Final concentration
Power SYBR Green master mix (2x)	10	1x

Primer (telomere-fwd (2 μM))	1	0.1 μM

Primer telomere-rev (2 μM)	1	0.1 μM

H_2_0	4	

DNA (5 ng/μl DNA)	4	20 ng total

a. For example a 96-well plate set up is described in Table 3

**Table 3:** Example of reaction plate set up using 24 samples.

	1	2	3	4	5	6	7	8	9	10	11	12
**A**	1	1	1	9	9	9	17	17	17	S1	S1	S1

**B**	2	2	2	10	10	10	18	18	18	S2	S2	S2

**C**	3	3	3	11	11	11	19	19	19	S3	S3	S3

**D**	4	4	4	12	12	12	20	20	20	S4	S4	S4

**E**	5	5	5	13	13	13	21	21	21	S5	S5	S5

**F**	6	6	6	14	14	14	22	22	22	S6	S6	S6

**G**	7	7	7	15	15	15	23	23	23	POS	POS	POS

**H**	8	8	8	16	16	16	24	24	24	NTC	NTC	NTC

All numbers refer to replicates of an individual sample; POS = positive control; 1301 cell line DNA; NTC = no template control; S1 = TEL STD A (= 1.18 × 10^8^); S2 = TEL STD B (= 1.18 × 10^7^); S3 = TEL STD C (= 1.18 × 10^6^) etc...

31. Mix well and briefly centrifuge

32. Pipette required amount (16 ul in our example) of master mix into each well of the PCR plate

a. The power SYBR Green has a high detergent content so pipette master mix carefully to avoid formation of unwanted bubbles in the master mix

33. Remove from PCR set-up hood

34. Pipette required volume of DNA into all sample wells

a. TAKE CARE - this pipetting step must be done with great care and precision

35. Pipette required volumes of standards, positive control and water for NTC into their respective wells.

36. Seal plate with optical clear film

37. Centrifuge briefly

38. Pause Point

a. Samples can be stored at 4degC O/N if required

#### Running the PCR (Timing: 2 hrs)

39. Turn on PCR machine

40. Input sample identifiers on qPCR running software, including NTC, positive control and quantities of standards (i.e. TEL STD A = 1.18 × 10^8 ^kb telomere..etc)

41. Cycling conditions (for both telomere and *36B4 *amplicons) are: 10 min at 95°C, followed by 40 cycles of 95°C for 15 sec, 60°C for 1 min, followed by a dissociation (or melt) curve.

42. Once PCR has completed - remove plate and discard

#### QC of results (TIMING 0.5 hrs)

43. Check for appropriate amplification in positive control and no signal in NTC

44. Observe amplification in standards and samples

**45. Setting baseline**. Because TEL STD A will amplify very early (at approx. Ct 5) you may need to alter background parameters on the qPCR output window.

46. **Standard curve**. Check standard curve by using results from TEL STD A dilutions. Using these concentrations you should observe the linear range of the reaction.

47. Ensure all target samples fall within the linear range - any samples that amplify outside this linear range should be removed from further analysis.

48. Check replicates for variation. Individual samples are analysed in triplicate and accepted only if the standard deviation of the Ct values are <1Ct (CV > 5%). It is expected that 90% of samples analysed will meet this criterion. Samples with differences in standard deviation greater than 1 Ct values should be removed from further analysis and re-analysed.

49. After amplification is completed the AB software produces a value for each reaction that is equivalent to kb/reaction based on the telomere standard curve values.

#### qPCR for SCG

50. Repeat from step 30 with single copy gene primers in reaction.

a. This value generate from SCG will be number of genome copies per reaction

#### Processing and analysing data

51. Export values (kb/reaction for telomere and genome copies/reaction for SCG) to csv format

52. The kb/reaction value is then used to calculate total telomere length in kb per human diploid genome.

53. The telomere kb per reaction value is divided by diploid genome copy number to give a total telomeric length in kb per human diploid genome.

54. OPTIONAL: This value can be further used to give a length per telomere by dividing by 92 (92 is the total number of telomeres on 23 pairs of chromosomes found in normal human cells).

### QC and comparison with other methods for measuring telomere length

DNA from the 1301 lymphoblastic cell line can be used as a long telomere control (telomere length of 70 kb) in each plate run. The inter- and intra- experimental coefficient of variation of the 1301 telomere length measurement by absolute qPCR should be less than 7% and 2%, respectively.

**Figure 2:**
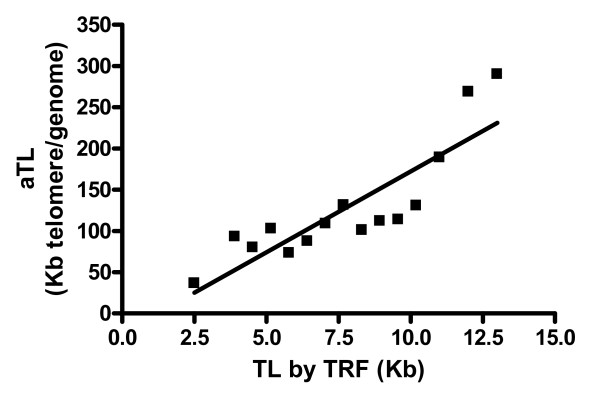
**Comparison of telomere length measurement methods**. Graph represents correlation between the TRF and aTL methods. Telomeres were measured in whole-blood, lymphocytes, mid-rectal biopsies and low and high controls (Telo-assay, Roche) (r^2 ^= 0.75, p < 0.0001).

The performance of the qPCR method for aTL it can be tested by comparison to the gold standard of telomere length measurements, Terminal Restriction Fragment analysis (TRF) [32]. Telomere lengths are determined by a TRF diagnostic kit (Roche Diagnostics, Australia). For example our studies demonstrated a strong correlation between results for TRF and the qPCR method for aTL (r^2 ^= 0.75, p < 0.0001) (Figure 2). The TRF measurement for the 1301 B-cell derived lymphoblastoid cell line, commonly used in Flow-FISH analysis of telomere length was reported to be 80 kb [33]. Using the absolute Real-Time PCR method, we measure the average telomere length for 1301 at 70 kb.

**Figure 3:**
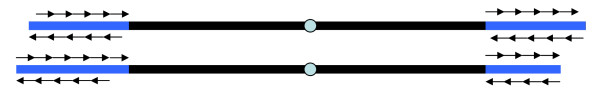
**Schematic representation of aTL measurement by qPCR**. One pair of chromosomes is shown, circles represent centromeres. Regions containing telomere repeats (TTAGGG)n are coloured blue. As shown, telomere lengths can vary between chromosomes and even between the two ends of a single chromosome. The arrows represent the primer pairs used to amplify the telomere sequences. The telomere PCR signal is a measure of telomere length, because the number of telomere primers that can bind the telomeric DNA at the beginning of the PCR is directly proportional to the total summed length of all the telomeres in the cell (adapted from [38]).

Telomere lengths, in different cell types, showed good agreement between the reported TRF values and the measures we obtained by the absolute qPCR method (Figure 2). However, there is a consistent discrepancy between the values obtained by the two methods with the TRF value being approximately 7 kb greater than that observed with the aTL[33-35]. It is recognised that the TRF method tends to overestimate telomere length because there is a considerable, highly variable, non-telomeric DNA component within TRFs [36]. In contrast, aTL only detects and amplifies intact TTAGGG sequences (Figure 3). In addition to TTAGGG, terminal restriction fragments in human DNA contain variable amounts of TTAGGG-like repeat sequences, which are detected as telomere sequence in the TRF assay. These include telomere repeat variants proximal to the telomere and the telomere adjacent sequences (reviewed in [36]). Additionally, as TRF is based on hybridisation the shorter the telomere the lower the hybridisation signal, consequently there is a telomere length threshold below which TRF analysis will not detect telomeric DNA. Although TRF analysis biases towards longer TL, this can be partially corrected by dividing the signal intensity by length in base pairs [37], although this is not always done.

## Trouble shooting advice

1. **Low quality DNA/low yield**. There are several possible causes for low yield/quality DNA. The most common reasons include inappropriate storage of sample following collection, non optimal starting amount (too much or too little) of sample collected, and insufficient cell lysis. **Suggestion**: Reisolate DNA from starting material.

2. **Low/no amplification in PCR (positive controls and standards)**. There are several possible causing for low/no PCR amplification. **Suggestion**: Check primer dilutions, you may need to set up fresh working dilutions of primers from oligomer stock.

3. Polymerase not working efficiently. **Suggestion**: Check PCR cycling to ensure appropriate activation of DNA polymerase is in place; select new aliquot of master mix and repeat PCR.

4. Fluorescent dye not working. **Suggestion**: Check PCR machine setting to ensure appropriate detection method for SYBR Green is selected; select new aliquot of master mix and repeat PCR.

5. Low/no amplification in PCR (samples).

a. low quality DNA (see above)

b. too much/too little DNA used (see Variation in Copy Number for expected results and modify accordingly)

6. **Amplification in NTC**. It is important that you determine the cause of this amplification; results from plates with NTC amplification should not be used. **Suggestion**: First check dissociation curve plot to determine likely causes of amplification in NTC; gDNA contamination or primer dimer formation. If cause of amplification in NTC is contamination (usually in the PCR set-up) change water and repeat NTC. Primer dimer formation is another cause of signal in the NTC wells. This amplification can be identified via examination of the dissociation curve; primer dimmer dissociation curves will appear very different to telomere dissociation profiles.

## Anticipated results

**Figure 4:**
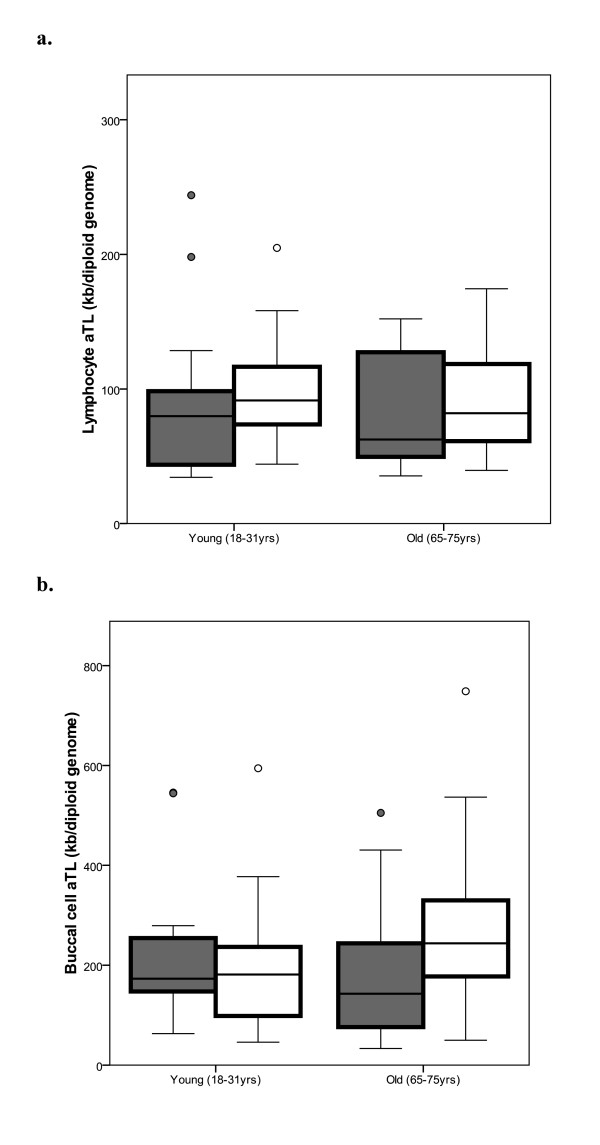
**Anticipated results of absolute telomere length in lymphocytes and buccal cells**. This cohort comprised 18 males (grey bars) and 25 females (open bars) in the young group (aged 18-31 years), and 25 males (grey bars) and 23 females (open bars) in the older group (65-75 yrs). Graph A shows the lymphocyte telomere length decreases with age in both males and females; Graph B shows buccal cell telomere length decreases with age in males, not in females. Data are shown as box plots which represent five-number summary of the data (the minimum, lower quartile, median, upper quartile and maximum).

A typical data set from lymphocytes and buccal cells of 18 males and 25 females in a young group (aged 18-31 years), and 25 males and 23 females in an older group (65-75 yrs) is shown in Figure 4. For the young group the lymphocytes had a mean aTL of 97.2 kb/diploid genome (range 35-260); the buccal cells had a mean aTL of 211.2 kb/diploid genome (range 45-594). The lymphocytes from the older group had a mean aTL of 86.6 kb/diploid genome (range 35-174); the buccal cells had a mean aTL of 230 kb/diploid genome (range 33-750).

## Competing interests

The authors declare that they have no competing interests.

## Authors' contributions

NJO conceived the method, designed and carried out the validation of the study and drafted the manuscript; MF was involved in the conception, design and supervision of the method development. All authors read and approved the final manuscript.
